# Ethics of AI in Radiology: A Review of Ethical and Societal Implications

**DOI:** 10.3389/fdata.2022.850383

**Published:** 2022-07-14

**Authors:** Melanie Goisauf, Mónica Cano Abadía

**Affiliations:** ELSI Services and Research, BBMRI-ERIC, Graz, Austria

**Keywords:** artificial intelligence, ethics, radiology, explainability, trustworthiness, bias

## Abstract

Artificial intelligence (AI) is being applied in medicine to improve healthcare and advance health equity. The application of AI-based technologies in radiology is expected to improve diagnostic performance by increasing accuracy and simplifying personalized decision-making. While this technology has the potential to improve health services, many ethical and societal implications need to be carefully considered to avoid harmful consequences for individuals and groups, especially for the most vulnerable populations. Therefore, several questions are raised, including (1) what types of ethical issues are raised by the use of AI in medicine and biomedical research, and (2) how are these issues being tackled in radiology, especially in the case of breast cancer? To answer these questions, a systematic review of the academic literature was conducted. Searches were performed in five electronic databases to identify peer-reviewed articles published since 2017 on the topic of the ethics of AI in radiology. The review results show that the discourse has mainly addressed expectations and challenges associated with medical AI, and in particular bias and black box issues, and that various guiding principles have been suggested to ensure ethical AI. We found that several ethical and societal implications of AI use remain underexplored, and more attention needs to be paid to addressing potential discriminatory effects and injustices. We conclude with a critical reflection on these issues and the identified gaps in the discourse from a philosophical and STS perspective, underlining the need to integrate a social science perspective in AI developments in radiology in the future.

## Introduction

Artificial Intelligence (AI) is seen as a promising innovation in the medical field. The term AI encompasses the ability of a machine to imitate intelligent human behavior (Tang et al., 2018). Machine learning (ML) is a subfield of AI which is widely applied to medical imaging (Pesapane et al., 2018a) and includes deep learning (DL), which produces data with multiple levels of abstraction (LeCun et al., 2015). These technologies have been developed to help improve predictive analytics and diagnostic performance, and specifically to improve their accuracy and ability to support personalized decision-making, as researchers have demonstrated that they can “outperform humans” when conducting medical image analysis (McKinney et al., 2020). Many researchers have also expressed the hope that they can help improve the provision of healthcare, and especially by enabling more rapid diagnosis, in coping with the workload resulting from an increase in screening (Mudgal and Das, 2020, 6), and in advancing health equity. Overall, AI systems are expected to have a significant impact in radiology.

“Artificial Intelligence (AI) is the talk of the town” (Ferretti et al., 2018, 320). Developments in AI are progressing rapidly in the medical field. This is reflected, for instance, in the enormous increase in publications on the development of AI systems in radiology, i.e., from about 100–150 per year in 2007–2008 to 700–800 per year in 2016–2017 (Pesapane et al., 2018a). However, the progress in development of the discourse on these technologies has not corresponded with the progress in the implementation of these technologies in healthcare. In other words, “The state of AI hype has far exceeded the state of AI science, especially when it pertains to validation and readiness for implementation in patient care” (Topol, 2019, 51). For example, few radiological AI systems have been implemented in the NHS, but several are awaiting approval (Mudgal and Das, 2020).

In the evolving field of Ethics of AI, investigations are carried out on the far-reaching consequences of AI in several areas of society. AI is steadily gaining importance in the medical field; as a result, researchers and practitioners are carefully considering the ethical and societal implications of AI use in order to avoid harmful consequences for individuals and groups, and especially those for the most vulnerable populations. Without a doubt, AI will have a profound impact in the field of radiology: It will affect end users and will introduce far-reaching challenges into clinical practice. While the introduction of AI is changing the role of the "radiologists-in-the-loop,” patients and other societal groups are being confronted with complex questions concerning the scope of informed consent, biases that may result in inequality, and risks associated with data privacy and protection, as well as open questions regarding responsibility and liability. These questions are accompanied by concerns that AI systems could perpetuate or even amplify ethical and societal injustices. Based on key ethical values such as respect, autonomy, beneficence, and justice (Beauchamp and Childress, 2001), several guiding principles and recommendations have been formulated to tackle these issues (Currie et al., 2020; Ryan and Stahl, 2020)—Such principles and recommendations have also been communicated on EU level (High-Level Expert Group on Artificial Intelligence, 2019), and initiatives such as FUTURE-AI (Lekadir et al., 2021) have been started, which have been developed to ensure that advances in AI systems and advances in AI ethics do not contradict one another.

In this paper, we contribute to the discourse on ethics of AI in radiology by reviewing the state-of-the-art literature and discussing the findings from a philosophical and social science perspective. We consider the comment made by (Mittelstadt and Floridi, 2016, 468), namely, that “reviewing literature is a first step to conduct ethical foresight, in the sense that it allows one to distinguish between issues and implications that are currently under consideration, and those that are not yet acknowledged or require further attention.” In our review, we highlight underexplored ethical and societal aspects and point out the necessary future research directions in the field. Our analysis was guided by two key research questions: (1) What types of ethical issues are raised by the use of AI in medicine and biomedical research, and (2) how are these issues being tackled in radiology, especially in the case of breast cancer? In the next section of this article, we describe the methods used, then present the outcomes of the review. We conclude the article with a critical discussion of the findings, highlight the identified gaps and indicate future directions.

## Methods

### Search and Eligibility Criteria

We performed a comprehensive review of ethical and societal issues that have already been identified and discussed, as well as how these issues have been addressed in the context of AI. To do so, we carried out a systematic review of state-of-the art academic literature between July and December 2021. Five search engines were used (Google Scholar, Microsoft Academic, PubMed, Scopus, and Web of Science) to identify relevant articles on these issues. Twelve search strings were created that included terms relevant to the research questions (e.g., “AI,” “ethics,” “radiology,” “imaging,” “oncology,” “cancer,” “predictive medicine,” “trustworthy,” “explainable,” “black box,” and “breast cancer”). Hence, the selection of the search terms aimed at including both key aspects in the general discussion of AI in medicine and biomedical research, as well as specific approaches for radiology and oncology. In addition, breast cancer was included in the search as a specific case to analyze in-depth the societal implications associated with social categories such as gender, race, and socioeconomic background. The different levels were expected to allow us to better situate the topic in the broader Ethics of AI discourse. “IT” was defined as an exclusion criterion to refine the search and limit it to the ethical and societal aspects related to AI. All search strings were applied to the five search engines using the “Publish or Perish” app, introducing some minor differences in punctuation to adapt to the internal logic of different search engines. The search was limited to articles written in English language and to papers published after 2017. Outputs consisted mainly of peer-reviewed journal articles, but also included literature in the form of commentaries, reports, and book chapters. These sources were not excluded from the sample, as they are also seen as contributing to the discourse on the ethics of AI use in radiology.

### Data Analysis

All identified records were imported into Microsoft Excel spreadsheets for further analysis. The subsequent screening procedure was conducted in two major steps. First, we scanned paper titles and abstracts to identify papers that included discussions on ethical and societal aspects of AI. Duplicates and papers that did not match the inclusion criteria were removed from the sample, as well as articles that were identified by the search engines because they contained an ethics statement. Second, the full texts of the resulting sample items (*n* = 56) were analyzed using thematic analysis (Terry et al., 2017).

Guided by our research questions, we coded each article in the final sample to develop overarching themes or patterns. Semantic codes were generated, on the one hand, to deductively assign terms also used in the search strings to the material (i.e., terms that are commonly used in the AI ethics discourse, such as “explainability,” “trustworthy,” and “black box”) and, on the other hand, inductively developed from the data. Reviewers coded independently on paper and by using the Atlas.ti software package. In the next analytical phase, the codes and coded text segments were collated to identify themes across the sample. Each theme and the corresponding text segments were analyzed to determine their specific content and depth, but also scrutinized to identify conceptual gaps.

## Results

**Figure 1:**
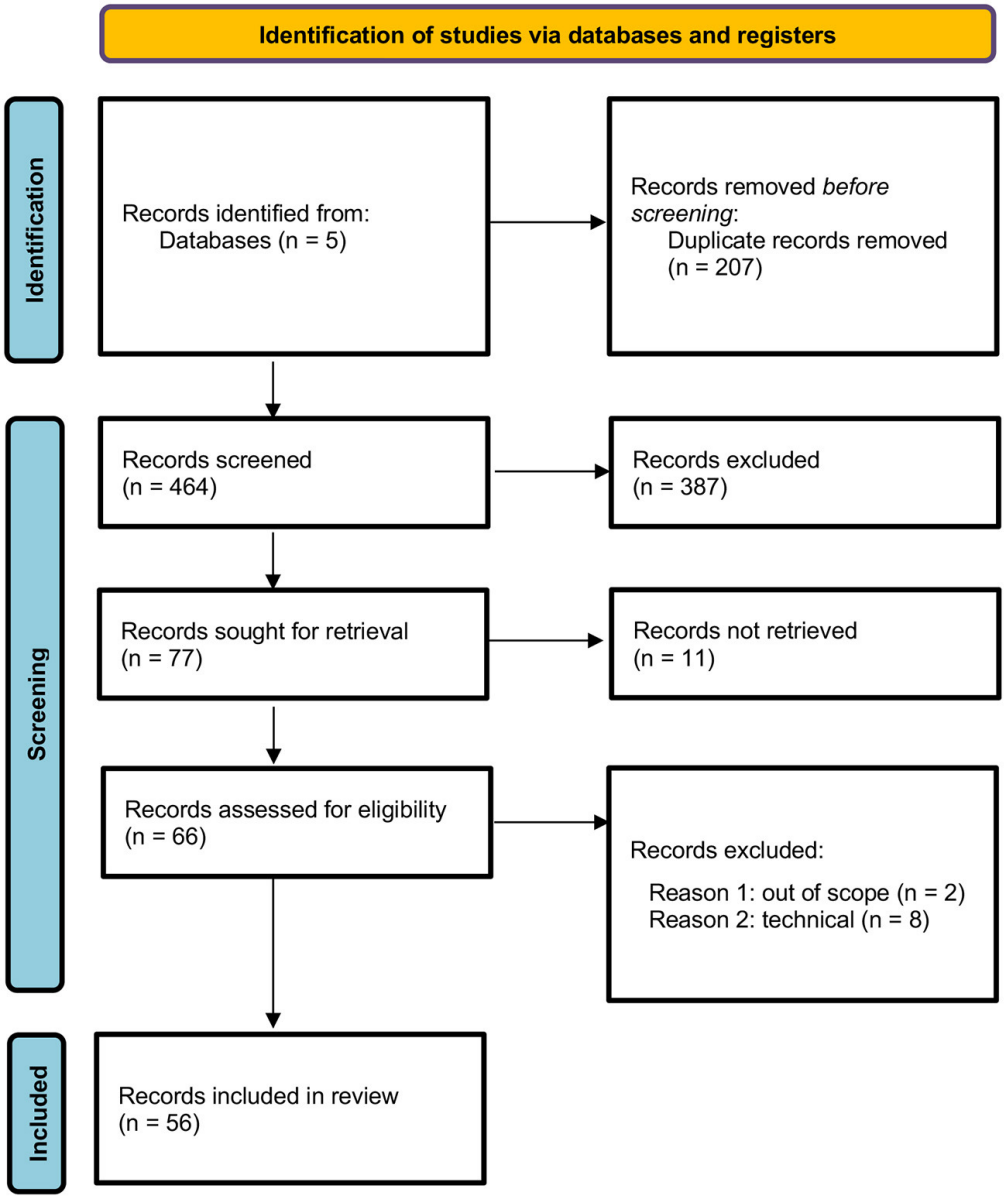
PRISMA flow diagram showing the results of the systematic literature review.

The results of the systematic literature review are included in an adapted PRISMA flow diagram (Figure 1) (Page et al., 2021).

After screening the records identified by the search engines to determine their eligibility for inclusion, the full texts of a final sample of 56 papers were reviewed. We observed that sources that placed an explicit focus on the ethical issues of AI and breast cancer were rare in the sample (two); some papers on breast cancer identified in the search were excluded, as these placed a primary focus on technical issues, were determined to be irrelevant for the aim of this study or were identified due to the presence of an ethics statement in the article. In terms of content, we concluded that no article in the sample placed an explicit focus on the ethics of AI use in radiology and breast cancer.

The review results show that the application of medical and radiological AI systems is widely discussed in the scientific discourse. As mentioned in the introduction, these technologies are accompanied by hypes and hopes regarding their potential to improve predictive analytics, diagnostic performance, and eventually patient outcomes, as well as challenges that arise due to the (potential) real-world application. During the analysis, certain topics were identified as especially important, which are mainly organized around approaches and principles. Guided by our research questions (i.e., what types of ethical issues are raised by medical AI and how these are tackled in radiology and the case of breast cancer in particular), we analyzed the key themes regarding their claims about ethical and societal implications.

In the next sections, we organize the key themes identified in the literature review as follows: First, we map the expectations regarding the application of AI systems in the medical field, as these are important indicators of their imagined innovative potential and how the discourse is framed, and then enumerate the key challenges. Second, we describe the ethical principles addressed in the literature, such as explainability, interpretability, trust/trustworthiness, responsibility and accountability, justice, and fairness. These sections are followed by a critical discussion of the ethical and societal implications of the results.

### Expectations

The analysis of the literature shows that the application of AI systems in healthcare has been welcomed, according to the expectations associated with this application. Thereby, we identified three main areas in which changes are expected, and especially areas in which improvements are expected: better analytical performance, benefits for patients, clinicians and society, and a change in the professional role of radiologists.

The expectation that AI will significantly improve diagnostics and patient care is a key assumption that is expressed throughout the sample. Choy et al. (2018) describe current AI applications to help with case triage, maximize image quality, detect and interpret findings automatically, perform automated processes related to treatment (e.g., in radiotherapy) and points out that these applications can support the personalization of treatment *via* predictive analytics by making scheduling easier. Hosny et al. (2018) identify three radiological tasks in which AI can play a significant role: in the detection of abnormalities, their characterization, and in monitoring changes. Other authors note that further applications of AI are expected to increase analytical power (i.e., to perform analyses more rapidly than humans and to minimize human error) and to identify as-yet unknown relationships (Pesapane et al., 2018a; Brady and Neri, 2020). Eventually, such applications can be used to detect diseases earlier and to provide proper treatment with fewer unnecessary procedures, better cost efficiency, and lower inter- and intra-reader variability (Mazurowski, 2020).

These advances in medical diagnostics are also expected to benefit the end users. For instance, Kelly et al. (2019) identify a quadruple aim for the application of AI systems in healthcare: improving experience of care, improving the health of populations, reducing per capita costs of healthcare, and improving the work life of healthcare providers. Ryan and Stahl (2020) highlight the ethical principle of beneficence and emphasize the supposition that AI should benefit societies and support the social as well as the common good.

The expectations regarding radiologists are ambiguous, with some scholars highlighting the fact that AI will outperform clinicians and be able to diagnose more rapidly and accurately. Bjerring and Busch (2021, 350) state “We can at least with some warrant adopt the assumption that AI systems will eventually outperform human practitioners in terms of speed, accuracy, and reliability when it comes to predicting and diagnosing central disease types such as cancer, cardiovascular diseases, and diseases in the nervous system.” On the other hand, some scholars consider that AI will not be able to perform all tasks that health practitioners currently do without any human intervention. Naqa et al. (2020) propose what they consider to be a realistic vision that keeps “humans-in-the-loop.” According to this perspective, AI systems will serve as physicians' partners, enabling them to deliver improved healthcare by combining AI/ML software with the best human clinician knowledge. This partnership would allow the delivery of healthcare that outperforms what either can deliver alone, thus improving both credibility and performance.

### Challenges

Despite the hype surrounding AI implementation in healthcare and radiology, numerous authors highlight the challenges this can raise. One key challenge concerns the data that are used to train AI, such as the lack of labeled (i.e., annotated) data. Massive amounts of data are needed to train algorithms (Matsuzaki, 2018) and training images must be annotated manually. This challenge is accompanied by a secondary challenge: the fact that the amount of radiological imaging data continues to grow at a disproportionate rate as compared to the number of available, trained readers (Hosny et al., 2018). While some authors propose using a model that has been developed to keep “humans-in-the loop,” others consider that the availability of human validation will limit the promises of AI. Tizhoosh and Pantanowitz (2018) comment that “The pathologist is the ultimate evaluation if AI solutions are deployed into clinical workflow. Thus, full automation is neither possible, it seems, nor wise as the Turing test postulates.”

The affordability of required computational expenses poses another challenge (Tizhoosh and Pantanowitz, 2018). Geis et al. (2019: 330) point out that AI could increase imbalances in the distribution of resources, creating a gap between institutions that have more and less “‘radiology decision-making' capabilities.” Small or resource-poor institutions may find it difficult to allocate the necessary resources to manage complex AI systems, especially those that are proprietary. These authors (Geis et al., 2019, 332) emphasize that “Almost certainly some radiology AI will be proprietary, developed by large academic or private health care entities, insurance companies, or large companies with data science expertise but little historical radiology domain knowledge. This may exacerbate disparities in access to radiology AI.”

The scarcity of resources is also closely connected to or could result in some form of bias, and in particular automation bias, which is the “tendency for humans to favor machine-generated decision, ignoring contrary data or conflicting human decisions” (Neri et al., 2020, 519). Geis et al. (2019, 332) argue that automation bias can lead to errors of omission, i.e., humans might fail to notice or might disregard the failure of AI tools. This could clash with the need identified in the literature to take a “human-in-the-loop” approach, as “risks may be magnified in resource-poor populations because there is no local radiologist to veto the results.” (Geis et al., 2019, 332).

Some doubts have been voiced in the literature regarding the possibility of implementing AI into daily clinical practice, as real-world deployments are still rare, and only a few algorithms have been clinically tested or implemented (Kelly et al., 2019; Mudgal and Das, 2020). In this regard, it is questioned if this implementation is a realistic goal and that it is not clear how to effectively integrate AI systems with human decision-makers (Tizhoosh and Pantanowitz, 2018). Other authors (Gaube et al., 2021) noted that, in the few cases where systems have been implemented, no proof of improved clinical outcomes has been provided, while Kelly et al. (2019) mention different challenges associated with the use of such systems, including logistical difficulties, quality control, human barriers, and algorithmic interpretability claims.

The challenges outlined so far highlight specific institutional and resource-related issues that may influence the further development of radiological AI; however, these issues also determine how ethical and social implications are reflected upon and manifested in addressing these challenges. This becomes tangible when examining the two major recurring themes identified in the reviewed sample: black box and bias.

#### Black Box

Many ML algorithms, and especially DL algorithms, are often referred to as operating in a “black box.” This black box is defined in the literature as “an apparatus whose inner-workings remain opaque to the outside observer” (Quinn et al., 2021, 2), as “oracular inference engines that render verdicts without any accompanying justification” (Watson et al., 2019, 2), or as “systems [that] are often unable to provide an audit trail for how a conclusion or recommendation is reached because of its convolutional nature” (Smith and Bean, 2019, 25). While some authors only indicate that black boxes generate challenges without going into further detail (Choy et al., 2018; Tizhoosh and Pantanowitz, 2018; Naqa et al., 2020; Kim et al., 2021), others have taken a more specific approach to address the consequences of applying black boxes in medicine. For instance, Bjerring and Busch (2021) apply Price's (2018) concept of black-box medicine: a subtype of AI-informed medicine where opaque or transparent AI systems play an essential role in decision-making. These definitions imply that opacity, intelligible justifications, and recommendations are key issues that need to be discussed when considering ethical requirements and the practitioner-patient relationship.

Ferretti et al. (2018) frame the problem of black boxes in medicine by applying the concept of opacity, which can be differentiated into three types: (1) lack of disclosure, (2) epistemic opacity, and (3) explanatory opacity. The (1) lack of disclosure is defined as a lack of transparency regarding the use of data. The patients' privacy and awareness of the use of their data, their consent (Mudgal and Das, 2020), and ownership of the data (Krupinski, 2020) appear as associated concerns. Larson et al. (2020) also address this issue, providing examples of partnerships between hospitals and data science companies that raised concerns about whether these companies are profiting from the use of patient data, often without their consent. Mudgal and Das (2020) also warn against the risks of defining the value of data on the basis of its face value. To mitigate this risk, “radiology's goal should be to derive as much value as possible from the ethical use of AI, yet resist the lure of extra monetary gain from unethical uses of radiology data and AI” (Geis et al., 2019, 330). To ensure the ethical use of data and to address a lack of disclosure, “patients should know who has access to their data and whether (and to what degree) their data has been de-identified. From an ethical perspective, a patient should be aware of the potential for their data to be used for financial benefit to others and whether potential changes in legislation increase data vulnerability in the future, especially if there is any risk that the data could be used in a way that is harmful to the patient” (Currie et al., 2020, 749). In this sense, regulations for safety, privacy protection, and ethical use of sensitive information are needed (Pesapane et al., 2018b). (2) Epistemic opacity is the lack of understanding of how the AI system works and, for Ferretti et al. (2018) it is caused by procedural darkness (the rules that the AI system is following are not available) or procedural ignorance (the rules are available, but it is impossible to understand them). (3) Explanatory opacity, on the other hand, is the lack of a clinical explanation: A system might find patterns that do not have a clinical explanation with the current medical knowledge.

Deep learning conflicts with ethical requirements: The lack of understanding and transparency regarding how an AI system reaches a decision presents a major ethical concern. However, a more explainable system diminishes the power of DL (Brady and Neri, 2020; Currie et al., 2020; Quinn et al., 2021). This conflict is related to the question of whether “high stakes” institutions, such as healthcare, should use black-box AI (Brady and Neri, 2020; Bjerring and Busch, 2021; Quinn et al., 2021). Bjerring and Busch (2021) note that AI introduces some obvious differences, but also point out that black boxes do not present a fundamentally new epistemic challenge, as opaque decision-making is already common in non-AI-based medicine. By keeping the “practitioner-in-the-loop,” however, at least some knowledge available to support informed decision-making. In the case of black-box medicine, “there exists no expert who can provide practitioners with useful causal or mechanistic explanations of the systems' internal decision procedures” (Bjerring and Busch, 2021, 17). Furthermore, some of the consequences of black-box medicine are epistemic in nature: Black-box medicine may lead to a loss of knowledge, and specifically to a loss of medical understanding and explanation and, thus, medical advances.

These challenges are associated with considerations about the impact of black-box AI on validity and the potential harm it presents patients. An opaque system makes it difficult to keep humans in the loop and enable them to detect errors and to identify biases. Such a system can have negative effects on underrepresented or marginalized groups and can also fail in clinical settings (Quinn et al., 2021). In addition, it can pose certain risks for radiologists, who are expected to validate something that they cannot understand (Neri et al., 2020), open them to adversarial attacks (Tizhoosh and Pantanowitz, 2018; Geis et al., 2019), or intensify the clash between black-box medicine and the duty of care, presuming that the radiologists have the ability to understand the technology, its benefits, and potential risks (Geis et al., 2019; Currie et al., 2020). The latter is also associated with depriving the patients of the ability to make decisions based on sufficient information and justifications, which contradicts the ethical requirement for the patients to exercise autonomy by giving their informed consent (Quinn et al., 2021). This type of medicine cannot be described as “patient-centered medicine” (Bjerring and Busch, 2021), and may have negative effects on the relationship of trust that is established between the patient and clinician.

#### Bias

The lack of transparency inherent in black-box AI tools is also a problem associated with bias. This lack is difficult to detect, measure, or correct unless the person using the tool has transparent access to the reasoning of the algorithm or the epistemic tools to understand this reasoning (Quinn et al., 2021). Radiology AI may also be biased by clinically confounding attributes such as comorbidities and by technical factors such as data set shifts and covariate shifts due to subtle differences in the raw and post-processed data that arise from the use of different scanning techniques (Geis et al., 2019), AI systems used in healthcare might have both a racial and a gender bias (Rasheed et al., 2021), but the reviewed literature discusses mainly racial bias. Many algorithms in medicine have been shown to encode, reinforce, and even exacerbate inequalities within the healthcare system (Owens and Walker, 2020) and can worsen the outcomes for vulnerable patients (Quinn et al., 2021). Such biases are introduced due to the data used to train an algorithm and the labels given to these data, which may be laden with human values, preferences, and beliefs (Geis et al., 2019). The generated outputs will thus eventually reflect social and political structures, including injustices and inequalities. Consequently, AI systems cannot provide entirely unbiased or objective outcomes based on incomplete or unrepresentative data; instead, they mirror the implicit human biases in decision-making (Balthazar et al., 2018; Pesapane et al., 2018b; Ware, 2018; Abràmoff et al., 2020). This has effects that extend beyond training, an aspect underlined by Quinn et al. (2021, 4), who point out that “most training data are imperfect because learning is done with the data one has, not the sufficiently representative, rich, and accurately labeled data one wants. […] even a theoretically fair model can be biased in practice due to how it interacts with the larger healthcare system.” According to Abràmoff et al. (2020) this “algorithmic unfairness” stems from model bias, model variance, or outcome noise. Model bias arises when models are selected to best represent the majority but not the unrepresented groups; model variance is caused by insufficient data from minorities, while outcome noise is caused by interference between unobserved variables and the model predictions. The latter can be avoided by broadening the scope of data to include underrepresented groups and minimize the possibility of unobserved variables interfering.

Common sources of bias that potentially promote or harm group level subsets are based on gender, sexual orientation, ethnic, social, environmental, or economic factors, as well as on unequal access to healthcare facilities and geographical bias. Referring to existing research, Owens and Walker (2020) and Quinn et al. (2021) point out racial bias that stems from the seemingly effective proxies for health needs (such as health costs) in algorithms that do not use race as a predictor for the models. Health costs are not a race-neutral proxies for health needs; this implies a need for a concerted and deep understanding of the social mechanisms of structural discrimination. Furthermore, biases in AI tools have a strong tendency to affect groups more strongly that are already suffering from discrimination based on these factors. Furthermore, AI biases have a strong tendency to affect groups more that are already suffering from discrimination based on these factors: “Blind spots in ML can reflect the worst societal biases, with a risk of unintended or unknown accuracies in minority subgroups, and there is fear over the potential for amplifying biases present in the historical data.” Kelly et al. (2019, 4) note that “Blind spots in ML can reflect the worst societal biases, with a risk of unintended or unknown accuracies in minority subgroups, and there is fear over the potential for amplifying biases present in the historical data.” The authors clearly illustrate this by providing the example of underperformance regarding the classification of images of benign and malignant moles on dark-skinned patients, because the algorithms are trained with data from predominantly fair-skinned patients. AI systems are often developed by companies in western countries and tested on Caucasian data, generating imbalances of representation in the datasets. “When the algorithm is trained on data that inherit biases or do not include under-represented population characteristics, existing disparities can be reinforced” (Akinci D'Antonoli, 2020, 504).

“Fairness and equality are not AI concepts” (Geis et al., 2019, 331). This statement indicates that AI tools cannot correct this type of bias on their own, but researchers developing such tools and companies providing such tools can. One solution described in the literature is to ensure diversity when collecting data and to address bias in the design, validation, and deployment of AI systems. Algorithms should be designed with the global community in mind, and clinical validation should be performed using a representative population of the intended deployment population. Careful performance analyses should be performed on the basis of population subgroups, including age, ethnicity, sex, sociodemographic stratum, and location. Understanding the impact of a new algorithm is particularly important; this means that, if the disease spectrum detected using the AI system differs from that identified using current clinical practice, then the benefits and harms of detecting this different disease spectrum must be evaluated (Kelly et al., 2019, 4–5). Owens and Walker (2020) emphasize the fact that making analyses “race neutral” is not enough and advocate taking a proactive, explicitly anti-racist approach; they even suggest that failing to recognize and anticipate structural bias in datasets or the social implications of AI systems should be considered as scientific misconduct. They urge readers to introduce a culture shift that would contribute to alleviating inequities stemming from unreflective algorithmic design. For this purpose, education on racial justice is needed at all levels, as researchers and providers often do not have the expertise to identify or address structural factors. Balthazar et al. (2018) suggest that active engagement with small population data sets is needed to consider social determinants of health and to promote access to data from underprivileged populations. Learning to identify these biases can promote “algorithmic fairness,” and ML approaches might be used to correct them (Abràmoff et al., 2020). Geis et al. (2019, 331) propose certain questions that can be asked to identify bias to advance toward algorithmic fairness: How and by whom are labels generated? What kinds of bias may exist in the datasets? What are the possible risks that might arise from those biases? What steps have we taken to mitigate these risks?

### Guiding Principles

Approaches that can be taken to meet the expectations described and to tackle the challenges are often formulated as principles in the literature. This reflects an understanding of “bioethics as a scholarly discipline and its methodological approaches, with focus on the so-called “principlism” and the widely known four principles, namely beneficence, non-maleficence, autonomy, and justice” (Rasheed et al., 2021, 15). The proliferation of guidelines and recommendations makes it difficult for developers and users of AI systems to decide which ethical issues to address (Ryan and Stahl, 2020). These principles are often developed to provide guidance for many different stakeholder groups and lack specificity, presenting concepts that are often too abstract and broad and are difficult to adopt to address practical issues (Ryan and Stahl, 2020).

#### Explainability and Interpretability

To manage the risks inherent in the use of medical black boxes and the resulting bias, the requirement is often posed that the way an AI system arrives at its decision must be transparent and sufficiently understandable for the “human-in-the-loop” to improve patient safety and to gain the patient's trust. For that reason, “explainability” has become a key principle in the area of AI ethics, and especially in the context of healthcare.

The discourse has developed such that explainability and interpretability have become two closely associated concepts, and these concepts are often used synonymously by different authors of the reviewed literature. However, these concepts express two different directions of thought: Interpretability refers to how well one can understand how an AI system works, while explainability refers to how well one can explain what happens in AI decision-making in understandable terms (Brady and Neri, 2020; Rasheed et al., 2021). The conceptual constellation revealed in the review of the literature overlaps, often without clarity, with the concepts of interpretability, explainability, intelligibility, understandability, transparency, trustworthiness, agency, accountability, reliability, explicability, communication, and disclosure. And some authors define one term by using another. For example, explainability is defined as “AI's capacity for transparency and interpretability” and “designing explainability into AI tools is essential if they are to be trusted and if their users are to be able to exercise agency when making decisions, whether they be professional or lay users. In other words, AI must be accountable to users for the ways in which they behave” (Procter et al., 2020, 2). In other papers, explainability is associated with transparency, as in the comment “if an algorithm fails or contributes to an adverse clinical event, one needs to be able to understand why it produced the result that it did and how it reached a decision. For a model to be transparent, it should be both visible and comprehensible to outside viewers. How transparent a model should be is debatable” (Geis et al., 2019, 331). And transparency is then related, in turn, to accountability, as illustrated by Akinci D'Antonoli's comment (2020, 509) that “Transparency and accountability principles can be brought under the explicability principle. Artificial Intelligence systems should be auditable, comprehensible and intelligible by “natural” intelligence at every level of expertise, and the intention of developers and implementers or AI systems should be explicitly shared.”

Overall, transparency is one of the most widely discussed principles in the AI ethics debate and is becoming one of the defining characteristics. Nevertheless, some scholars still question how much transparency AI systems should have without leaving them open to malicious attacks or intellectual property breaches (Ryan and Stahl, 2020) or enabling their misuse for harmful purposes outside the clinical context (Watson et al., 2019). Brady and Neri (2020) point out that the more explainable an AI model is, the less it can utilize the power of DL. Thus, some authors consider that transparency and explainability should be placed in a human context, as humans are often also unable to fully explain their decisions and the outcomes of their reasoning. Watson et al. (2019, 3) specifically mention that “clinicians are not always able to perfectly account for their own inferences, which may be based more on experience and intuition than explicit medical criteria.” Even without the intervention of AI, complex diagnoses can be difficult to explain to other professionals or to patients. Even without the intervention of AI, complex diagnosis can be difficult to explain to other professionals or to patients. If this perspective is taken, the expectation for AI should be that “AI can explain itself at least as well as human explain their own actions and reasonings, systems would demonstrate transparency and honesty” (Ware, 2018, 21).

The issue of interpretability and explainability has interesting ramifications with reference to contestability, which is understood as the capacity of individuals (patients or medical staff) to contest and counter medical decisions (Sand et al., 2021). In line with this, the European General Data Protection Regulation (GDPR) has emphasized the patient's right to receive an explanation as a top priority in ML research. The right to an explanation encompasses the right to receive an explanation about the outputs of the algorithm, especially when decisions need to be made that significantly affect an individual. Ferretti et al. (2018, 321) explain that “the idea of a right to explanation stems from the value of transparency in data processing and it is intended to counterbalance the opacity of automated systems.” Individuals have a right to protect themselves against discrimination; to do so, they have a right to know how decisions that affect them are made. In the case of AI applications in healthcare, individuals should have a right to contest (suspected) bias in the diagnostic process or the treatment selection process.

#### Trust and Trustworthiness

“Trust is such a fundamental principle for interpersonal interactions and is a foundational precept for society to function” (Ryan and Stahl, 2020, 74) and, thus, it is a key requirement for the ethical use of AI. As such, it has been chosen as one of the guiding principles by the High-Level Expert Group of the European Commission (2019) and identified as the defining paradigm for their ethics guidelines.

The review enabled us to find some consensus in the literature that black boxes and the lack of interpretability and explainability can lead to a lack of trust (worthiness) in and acceptance of AI systems by clinicians and patients (Ware, 2018; Quinn et al., 2021). This aspect requires special consideration, as AI involves an element of uncertainty and risk for the vulnerable patient. Therefore, explainability is key to encouraging trust in an AI system, i.e., because people trust what they can understand (Larasati and DeLiddo, 2020). Similarly, (Spiegelhalter, 2020, 8) connects trust with explainability when proposing a series of questions about trustworthiness that include “Could I explain how it works (in general) to anyone who is interested? Could I explain to an individual how it reached its conclusion in their particular case?” Transparency becomes a fundamental factor: AI systems should be transparent enough that those using them can have access to the processes that govern them and be able to explain them. This requires access to accessible, intelligible, and usable information that can be effectively evaluated. In turn, a lack of explainability, lack of transparency, and lack of human understanding of how AI systems work will inevitably result in clinicians failing to trust decisions made by AI, as well as failing to trust the reliability and accuracy of such systems (Larasati and DeLiddo, 2020; Bjerring and Busch, 2021).

Given the fact that trust is repeatedly emphasized in the literature as a key ethical principle and mentioned as a prerequisite for the successful implementation of AI systems in medical practice, it is surprising that the authors of the reviewed papers preserve a relative silence regarding the need for an in-depth analytical approach with trust as a concept, although they echo the value of such trust. However, Quinn et al. (2021, 3) note that “the medical profession is built on various forms of trust”—and these forms of trust, its conditions, and social and institutional contexts would require a deeper analysis.

#### Responsibility and Accountability

AI's lack of transparency also has an impact on matters of responsibility and accountability. Ryan and Stahl (2020, 74) specifically point out that “End users should be able to justly trust AI organizations to fulfill their promises and to ensure that their systems function as intended […]. Building trust should be encouraged by ensuring accountability, transparency and safety of AI.” In that sense, “criminal liability, the tort of negligence, and breach of warranty must be discussed before utilizing AI in medicine” (Matsuzaki, 2018, 268). Neri et al. (2020) pose the question of who is responsible for benefits and harms resulting from the use of AI in radiology, and, like Akinci D'Antonoli (2020), claim that radiologists remain responsible for the diagnosis when using AI, even if they might be validating something unknown that is based on black boxes and possible automation bias. Therefore, radiologists should be taught how to use AI tools appropriately and familiarized with the guiding principles for increasing trust in AI. (Geis et al., 2019, 333) underlined this point effectively by stating that “Radiologists will remain ultimately responsible for patient care and will need to acquire new skills to do their best for patients in the new AI ecosystem.”

Sand et al. (2021) argue that the kind of accountability and responsibility that is being pursued in medical AI is connected to liability and blame. As an alternative, they propose a “forward-looking responsibility,” which “can be understood as a safeguard to decrease the risk of harm in cases of cognitive misalignment between the physicians and the AI system—when an AI output cannot be confirmed (verified or falsified)” (Sand et al., 2021, 3). Accordingly, the authors list the following responsibilities of clinicians: the duty to report uncertainty (sensitivity/specificity rates) to the patients; to understand and critically assess whether AI outputs are reasonable given a certain diagnostic procedure; to know and understand the input data and its quality; to have an awareness of their own experience and decline in skills; to have an awareness and understanding of the specificity of the task; and to assess, monitor, and report the output development over time.

One of the challenges of AI application in healthcare is the role of private companies who own the AI systems. Ryan and Stahl (2020, 71) mention the risk that companies try to “obfuscate blame and responsibility.” This lack of transparency regarding who is truly responsible and accountable further complicates issues of liability and undermines the ability of clinicians to act with integrity. Mudgal and Das (2020, 7) note that this lack of transparency and the subsequent problems that arise could be solved by maintaining a “human-in-the-loop” perspective, keeping the liability and responsibility within the field of responsibility of the radiologist and their employer.

#### Justice and Fairness

Justice is one of the four principles of bioethics: autonomy, beneficence, non-maleficence, and justice (Beauchamp and Childress, 2001). Some of the reviewed sources refer to some extent to which these four principles apply to AI (Akinci D'Antonoli, 2020; Currie et al., 2020; Rasheed et al., 2021). Justice is also one of the three principles proposed in the Belmont Report (United States National Commission for the Protection of Human Subjects of Biomedical Behavioral Research, 1978), one of the most widely recognized standards for biomedical ethics. In this report, justice refers to the idea that the benefits and costs of research and medical care should be distributed fairly (Larson et al., 2020).

Along with trust, transparency, accountability, and other principles, “diversity, non-discrimination and fairness” are principles that were proposed by the High-Level Experts Group on Artificial Intelligence of the European Commission in 2018. As Neri et al. (2020, 519) state, “the group recommended that the development, deployment and use of AI systems should adhere to the ethical principles of respect for human autonomy, prevention of harm, fairness/equity and explicability.” The principle of justice often appears to be associated with beneficence and non-maleficence, as the unfair distribution of resources leads to discrimination and can cause harm. (Geis et al., 2019, 330) pointed out that it is necessary to “inspire radiology AI's builders and users to enhance radiology's intelligence in humane ways to promote just and beneficial outcomes while avoiding harm to those who expect the radiology community to do right by them.” The association between injustice, discrimination, and unfair decisions made by AI systems has been also linked to bias in the reviewed literature, as “discrimination and unfair outcomes stemming from algorithms has become a hot topic within the media and academic circles” (Ryan and Stahl, 2020, 67). Biased AI systems lead to unfair, discriminatory behavior or mistaken decisions (Morley et al., 2020) and to the aforementioned “algorithmic unfairness” (Abràmoff et al., 2020).

Integrating AI systems in medicine incurs the risk of replicating discriminations that already exist in society; therefore, “the development of AI should promote justice while eliminating unfair discriminations, ensuring shareable benefits, and preventing the infliction of new harm that can arise from implicit bias” (Akinci D'Antonoli, 2020, 508–509). AI tools can decide in favor of one group of patients due to implicit biases rather than prioritizing a real emergency in radiology, underlining the necessity for everybody involved in the process to adhere to ethical guidelines that promote justice.

## Discussion

This literature review was carried out to identify ethical issues discussed in the recent academic literature associated with the use of AI in healthcare and to determine how these are being tackled in view of biomedical research, and especially in radiology and oncology imaging. This review enabled us to identify key themes which place a focus on expectations about medical AI, challenges posed by the use of this technology, and approaches that can be taken to ensure ethical AI use. Most of these themes are formulated by the authors as principles. In this section of this article, we critically discuss our findings from an ethical and social science perspective.

Several expectations are expressed in the literature regarding the potential for medical AI use to improve diagnostic performance and patient outcomes, but the socio-technological conditions under which these expectations can be met, and, at the same time, challenges can be managed are not clearly defined. We previously quoted that “the state of AI hype has far exceeded the state of AI science, especially when it pertains to validation and readiness for implementation in patient care” (Topol, 2019, 51). This statement illustrates an important gap: The contexts in which medical AI tools are being implemented have not been thoroughly explored. Considering the results of our review, this holds particularly true regarding the close connection between AI algorithms and societal structures. Although some scholars have discussed the fact that AI use “can increase systemic risks of harm, raise the possibility of errors with high consequences, and amplify complex ethical and societal issues” (Geis et al., 2019, 330), few studies have clearly defined exactly how AI tools interact with pre-existing systemic harm, how they can contribute to this harm, or how complex ethical and societal issues might be amplified through the use of such tools. In the reviewed literature, we identified a need for profound, specific, and interdisciplinary conversations about how firmly AI is embedded in systemic structures and power relations that intersect with identity traits (e.g., gender, race, class, ability, education) and about the implications of private ownership and the role of corporations, profit-making, and geopolitical structures.

### Bias

In that sense, we have observed that bias has not been framed in the context of power relations and societal conditions, nor has it been referenced to the existing body of research on, e.g., how gender and race shapes and affects biomedicine and healthcare practice (Roberts, 2008; Schiebinger and Schraudner, 2011; Oertelt-Prigione, 2012; Kaufman, 2013) or how gender and racial bias in algorithms could have a negative impact in certain areas of society (e.g., O'Neil, 2016; Noble, 2018). Bias has been shown to affect every stage of data processing (i.e., in generating, collecting, and labeling data that are used to train AI tools) and to affect the variables and rules used by the algorithms. Hence, AI tools can be taught to discriminate, reproduce social stereotypes, and underperform in minority groups, an especially risky proposition in the context of healthcare (Char et al., 2018; Wiens et al., 2019).

In the analyzed sample, little attention was given to sex and gender bias in AI systems used in healthcare. Nonetheless, research has already been done to analyze in detail how sex and gender bias is generated, how it affects patients and society, and how its effects can be mitigated. Using sex- and gender-imbalanced datasets to train deep-learning-based systems may affect the performance of pathology classification with minority groups (Larrazabal et al., 2020). Other authors also show that these social categories could influence the diagnosis although there is no direct link to the disease, and that potentially missed detection of breast cancer at mammography screening was greater among socioeconomically disadvantaged groups (Rauscher et al., 2013). Unfortunately, most of the currently used biomedical AI technologies do not account for bias detection, and most algorithms ignore the sex and gender dimensions and how these contribute to health and disease. In addition, few studies have been performed on intersex, transgender, or non-binary individuals due to narrow and binary background assumptions regarding sex and gender (Cirillo et al., 2020). Ignoring how certain identity traits affect the application of AI systems in healthcare can lead to the production of skewed datasets and harm certain minority people and groups. Applying feminist standpoint theory (Haraway, 1988; Hekman, 1997), some authors argue that all knowledge is socially situated and that the perspectives of oppressed groups are systematically excluded from general knowledge and practices that ignore the specific identity traits of certain individuals. Based on this argument, knowledge must be presented in a way that enables people to be aware of intersecting power relations that influence its production. The results of our literature review indicate that, rather than ignoring sex, gender, or race dimensions, close attention must be paid to these dimensions in datasets (Zou and Schiebinger, 2018; Larrazabal et al., 2020), even to the extent of introducing an amount of desirable bias to counteract the effects of undesirable biases that result in unintended or unnecessary discrimination (Cirillo et al., 2020; Pot et al., 2021).

Diversity in the datasets becomes an increasingly important point that is being addressed by researchers to counteract bias that can be potentially harmful (Leavy, 2018). Nonetheless, ensuring diversity in and of itself is not enough (Li et al., 2022); more research is needed to understand how discrimination intersects with socioeconomic factors to keep bias from being introduced into healthcare algorithms through structural inequalities in society (Quinn et al., 2021). Anticipating structural bias in datasets and understanding the social implications of using AI systems before their implementation is considered best practice; some authors in the sample even propose that failing to do so should be qualified as scientific misconduct (Owens and Walker, 2020). This will require reflecting on how social categories are constructed in big data-driven research and on how the underlying social classification and categorization systems are incorporated into and reproduced in the knowledge produced from analyzing the existing datasets (Goisauf et al., 2020).

### Lack of Analytic Accuracy

We observed that explainability and interpretability were often used interchangeably with other terms such as understandability and even transparency in our sample, as clear definitions of and analytic distinction between the terms are lacking. The lack of analytical precision that can be observed in the ethics of AI literature often leads to a lack of specificity and vague assumptions that do not enable scholars to reach the core of certain issues that are associated with epistemic justice (Fricker, 2007). The GDPR, for instance, states that subjects have a right to understand their lived experiences, especially experiences of injustice. Although research addresses the problem of how this right to an explanation is outlined in the legislation (Edwards and Veale, 2017), we argue that the lack of knowledge about why and how certain decisions that impact (negatively) our lives are made constitutes a specific wrongful act, i.e., epistemic injustice (Fricker, 2007). This injustice results in someone being wronged specifically in their capacity as a possessor of knowledge; they are wronged, therefore, in a capacity essential to human value. The opacity of AI and the implications of the use of AI tools makes it difficult for patients to exercise their autonomy. This inability is consequently also reflected in their practical limitation to give their informed consent and affects their capacity to contest decisions. To address epistemic injustices, knowledge must be made available to people affected by the decisions made by AI technology.

In our sample literature, the possibility of making information available and understandable is often treated as a technical feature of AI. It may then seem as though these issues are technical problems that can be solved by applying technical solution that deal with black boxes. Again, we have observed a need to take a social sciences perspective and to achieve a broader understanding of how our epistemic capabilities are also intertwined with power relations. In “AI ethics, technical artifacts are primarily seen as isolated entities that can be optimized by experts so as to find technical solutions for technical problems. What is often lacking is a consideration of the wider contexts and the comprehensive relationship networks in which technical systems are embedded” (Hagendorff, 2020, 103). It will be necessary to carefully consider the structures that surround the production and distribution of knowledge by performing further analyses of the ethics of AI in healthcare.

### Trust

Trust was often mentioned as an important factor in the reviewed literature, and trustworthiness has become a key principle regarding ethical AI. As we have shown, a clear definition and deeper understanding of the complexities of trust in AI are lacking. In the reviewed literature, for example, we found that trustworthiness is conflated with acceptance (Gaube et al., 2021) or explainability (Larasati and DeLiddo, 2020). Some authors have mentioned that “a possible imbalance in the data should be considered when developing the model to ensure the trustworthiness of the model” (Alabi et al., 2020, 7). However, for a model to be considered worthy of trust, more than simple technical solutions that even out technical “imbalances” in the training phase are needed, and especially when a risk of gender or racial bias exists. This is a more complex issue that will need to be addressed. Also, while it is important to encourage trust in technology, trust is built on the foundation of social relations. Healthcare practitioner-patient relationships are based on trust and empathy (Morley et al., 2020), and decision-making in the medical context, and especially in connection to technology, is often based on “gut feelings” (Goisauf and Durnová, 2018).

Previous research has shown that trust cannot be understood as unidirectional. Instead, trust needs to be understood as a complex, situated, context-dependent, and relational concept that involves several trustor/trustee relationships, such as trust in persons (e.g., scientists who trust each other, patients who trust scientists and clinicians), technology, and institutions (Wyatt et al., 2013; Bijker et al., 2016). Trust involves “the willingness to accept vulnerability based on positive expectations about another's intentions or behaviors […] Trust makes decision making more efficient by simplifying the acquisition and interpretation of information. Trust also guides action by suggesting behaviors and routines that are most viable and beneficial under the assumption that the trusted counterpart will not exploit one's vulnerability” (McEvily et al., 2003, 92–93). In building trust, embodied experience matters, and this experience occurs as an emotional reaction, e.g., in the form of the aforementioned “gut feelings” (Goisauf and Durnová, 2018). Trust or more precisely trusting relationships are fragile and require continuous work, which means that they need to be actively established and sustained. This includes trustworthiness (i.e., the idea that a person or object is worthy of being trusted), which is a key requisite for the sustainability of a trusting relationship (McEvily et al., 2003). To ensure trustworthiness, researchers must understand how trusting relationships are constituted *via* the social process, how trust in technologies is established and sustained, and under what conditions AI can be deemed trustworthy.

This discussion places an emphasis on trusting relationships between a practitioner and patient regarding medical AI use, the expectations and brings the needs of these actors into focus. Unfortunately, this is rarely the case in the reviewed literature, as relatively little attention is paid to the patients' and radiologists' perspectives, with only a few exceptions (e.g., Balthazar et al., 2018). However, (Ferretti et al., 2018, 331) stated that “more research is needed to understand patients' and physicians' attitudes toward opacity in AI systems.” Patients clearly want to be informed about how their health data are used (also a requirement of the GDPR), and the engagement of members of the public, patients, practitioners, and those developing the technology will be crucial to build trust and ensure both public and professional support.

## Conclusions and Future Directions

Performing this literature review, we have looked back at how current discourses revolve around the ethical and societal issues related to AI use in radiology. We have identified imaginaries of science and technology as aspects that are neutral, universal, and detached from societal structures, imaginaries that have already been described in the philosophy of science and STS fields (Haraway, 1988; Longino, 1990; Fox Keller, 1996).

We have observed that the current literature discourse does not delve into the broader origins and implications of bias, especially when bias is treated only as a technical problem with a technical solution. We believe that integrating a social science perspective into the analysis of ethical and societal issues associated with AI use in radiology is crucial to understanding the scope of these issues. To thoroughly address the topic of ethical AI use in radiology, a perspective must be taken to analyze how science is situated in a certain socioeconomic context and to understand the application of AI systems in medicine as a situated practice. Understanding the socioeconomic context is a fundamental step that will enable scholars to gain this perspective. In the future, inter- and trans-disciplinary research should be carried out to help situate knowledge production and its ethical and societal implications. In this sense, it will be necessary to shift from DL about to a deep understanding of the societal implications, and in particular to an understanding of the interactions of social values and categories with scientific knowledge production, of the relations between knowledge and societal trust that affects how science functions in society, and especially of how new technologies are perceived and accepted in society.

This review and the ensuing discussion also enabled us to identify a lack of precision regarding the use of terms for principles that have been proposed to apply AI technology more ethically in the future. Terms such as trustworthiness, transparency, or trust are extensively used in the literature, often without clearly defining specifically how they are meant or used. Researchers working in the field of ethics of AI in medicine will need to strive for accuracy and precision by providing clear definitions for these concepts in this specific context and by situating them within a broader context. In order to do this, interdisciplinary research with social scientists but also with clinicians in order to incorporate clinical concepts (Lekadir et al., 2021, 31) will be crucial.

More interdisciplinary and concrete research will deepen our understanding of biases in radiology. Adopting an intersectional perspective that takes into consideration how different traits of our identity intersect will be crucial, especially in the case of breast cancer. As previous research has shown, other factors that intersect with gender contribute to the formation of bias, such as ethnicity, skin color, socioeconomics, geography or breast density (Lekadir et al., 2021). In this regard, the issue of gender bias in female-only datasets requires a more detailed analysis. Considering breast cancer in connection to gender can lead to the abridged conclusion that gender bias could not have a significant impact. However, this reflects a one-dimensional understanding of gender as a social category, since gender is never isolated, but occurs at the intersection with other categories. Therefore, women cannot be assumed to be a homogeneous group, but are differentiated along other categories such as age, race, and socioeconomic background, which, as has been shown, could have an influence on breast cancer diagnosis.

In conclusion, the value of AI for radiology would increase by integrating a more precise and interdisciplinary consideration of the societal context in which AI is being developed to generate more just outcomes and allow all members of society equal access to the benefits of these promising applications.

## Data Availability Statement

The original contributions presented in the study are included in the article/supplementary materials, further inquiries can be directed to the corresponding author/s.

## Author Contributions

All authors listed have made a substantial, direct, and intellectual contribution to the work and approved it for publication.

## Funding

This study was funded by EuCanImage, a project that has received funding from the European Union's Horizon 2020 research and innovation program under grant agreement no. 952103.

## Conflict of Interest

The authors declare that the research was conducted in the absence of any commercial or financial relationships that could be construed as a potential conflict of interest.

## Publisher's Note

All claims expressed in this article are solely those of the authors and do not necessarily represent those of their affiliated organizations, or those of the publisher, the editors and the reviewers. Any product that may be evaluated in this article, or claim that may be made by its manufacturer, is not guaranteed or endorsed by the publisher.
